# Association of Chronic Lower Respiratory Disease With County Health Disparities in New York State

**DOI:** 10.1001/jamanetworkopen.2021.34268

**Published:** 2021-11-29

**Authors:** Yu-Che Lee, Ko-Yun Chang, Sanjay Sethi

**Affiliations:** 1 Department of Medicine, University at Buffalo–Catholic Health System, Buffalo, New York; 2 Division of Chest Medicine, Taichung Veterans General Hospital, Taichung, Taiwan; 3 Division of Pulmonary, Critical Care and Sleep Medicine, Department of Medicine, Jacobs School of Medicine and Biomedical Sciences, University at Buffalo, Buffalo, New York

## Abstract

**Importance:**

Chronic lower respiratory disease (CLRD) is the fourth leading cause of death in the United States, which imposes a considerable burden on individuals, families, and societies. The association between county-level health disparity and CLRD outcomes in New York state needs investigation.

**Objective:**

To evaluate the associations of CLRD outcomes with county-level health disparities in New York state.

**Design, Setting, and Participants:**

In this cross-sectional study, CLRD age-adjusted hospitalization for 2016 and mortality rates from 2014 to 2016 were obtained from the New York state Community Health Indicator Reports provided by the New York state Department of Health. County Health Rankings were used to evaluate various health factors to provide a summary *z* score for each county representing the county health status and how that county ranks in the state. Data analysis was performed from November 2020 to March 2021.

**Main Outcomes and Measures:**

The main outcomes were age-adjusted hospitalization and mortality rates for CLRD. The *z* score was calculated from the County Health Rankings, which includes subindicators of health behaviors, clinical care, social and economic factors, and physical environment. Pearson *r* and linear regression models were estimated.

**Results:**

During the study, 60 335 discharges were documented as CLRD hospitalizations in 2016 and 20 612 people died from CLRD from 2014 to 2016 in New York state. After adjusting for age, the CLRD hospitalization rate was 27.6 per 10 000 population, and the mortality rate was 28.9 per 100 000 population. Among 62 counties, Bronx had the highest hospitalization rate (64.7 per 10 000 population) whereas Hamilton had the lowest hospitalization rate (6.6 per 10 000 population). Mortality rates ranged from 17.4 per 100 000 population in Kings to 62.9 per 100 000 population in Allegany. County Health Rankings indicated Nassau had the lowest *z* score (the healthiest), at −1.17, but Bronx had the highest *z* score (the least healthy), at 1.43, for overall health factors in 2018. An increase of 1 point in social and economic factors *z* score was associated with an increase of 17.6 hospitalizations per 10 000 population (β = 17.61 [95% CI, 10.36 to 24.87]; *P* < .001). A 1-point increase in health behaviors *z* score was associated with an increase of 41.4 deaths per 100 000 population (β = 41.42 [95% CI, 29.88 to 52.97]; *P* < .001).

**Conclusions and Relevance:**

In this cross-sectional study, CLRD outcomes were significantly associated with county-level health disparities in New York state. These findings suggest that public health interventions and resources aimed at improving CLRD outcomes should be tailored and prioritized in health disadvantaged areas.

## Key Points

**Question:**

Are county-level health disparities associated with chronic lower respiratory disease (CLRD) outcomes in New York state?

**Findings:**

In this cross-sectional study including 60 335 CLRD hospitalizations and 20 612 CLRD deaths in 62 counties in New York state, county-level health indicators as assessed by County Health Rankings were significantly associated with CLRD hospitalization and mortality rates.

**Meaning:**

These findings suggest that public health interventions and resources aimed at improving CLRD outcomes should be tailored and prioritized in health disadvantaged areas.

## Teaser

This cross-sectional study evaluates the associations of county-level health disparities with chronic lower respiratory disease hospitalization and mortality outcomes in New York state.

## Introduction

Chronic lower respiratory disease (CLRD) is defined by the Centers for Disease Control and Prevention (CDC) and the World Health Organization as encompassing 4 major diseases: chronic obstructive pulmonary disease (COPD), chronic bronchitis, emphysema, and asthma.^1,2,3^ In the United States, approximately 14.8 million people have been diagnosed with COPD and more than 25 million people have asthma.^4,5^ According to the CDC, CLRD has resulted in 47.8 deaths per 100 000 population, making it the fourth leading cause of death in 2019.^6^ As a major public health issue, CLRD imposes a considerable burden on individuals, families, and societies. In 2008, the direct cost for health care expenditures related to CLRD was estimated at $53.7 billion and the indirect cost due to lost productivity was estimated at $14.3 billion.^4^

Health behaviors and social determinants of health have been identified as the most important modifiable risk factors associated with CLRD, which include tobacco smoking, indoor and outdoor air pollution, exposure to allergens and occupational agents, unhealthy diet, obesity, and physical inactivity.^7^ Even though several studies clearly indicated socioeconomic status (SES) and health disparities could contribute to CLRD outcomes at regional or national level, to our knowledge, no study has investigated the association between county-level health disparity and CLRD outcomes in New York state. In this study, we hypothesized that CLRD hospitalizations and mortality would be significantly correlated with overall county health status and county-level health indicators. Our aim was to examine and provide a general overview of the association between CLRD outcomes and health disparities in New York state.

## Methods

This cross-sectional study was deemed exempt from review and informed consent by the University at Buffalo institutional review board because this study only involved analysis of deidentified data in the public domain. This study followed the Strengthening the Reporting of Observational Studies in Epidemiology (STROBE) reporting guideline.

### Data Resources and Study Design

The CLRD age-adjusted hospitalization rates per 10 000 population for 2016 and mortality rates per 100 000 population for 2014 to 2016 were obtained from the New York state Community Health Indicator Reports provided by the New York State Department of Health. The *International Statistical Classification of Diseases, Tenth Revision, Clinical Modification* (*ICD-10-CM*)^8^ codes used for CLRD are codes J40-J47. The standard population used for age adjustment was the 2000 US population.

The County Health Rankings (CHR) program was created as a collaboration between the Robert Wood Johnson Foundation and the University of Wisconsin Population Health Institute. The CHR evaluates various health factors to provide a summary score for each county representing how that county ranks in the state. The county overall health factors rankings are calculated based on a weighted sum of *z* scores for each health factor in 4 categories: 30% for health behaviors (ie, tobacco use, diet and exercise, alcohol and drug use, and sexual activity), 20% for clinical care (ie, access to care and quality of care), 40% for social and economic factors (ie, education, employment, income, family and social support, and community safety), and 10% for physical environment (ie, air and water quality, housing, and transit). The choice to determine weights for each of the measures was based on its relative importance and guided by 5 perspectives, including historical perspective, review of the literature, weighting schemes used by other health rankings, analytic approach, and pragmatic approach involving stakeholder engagement.^9,10^ The CHR data are gathered from existing surveillance methods, such as the Behavioral Risk Factor Surveillance System, CDC, National Center for Health Statistics, US Department of Education, Bureau of Labor Statistics, and American Community Survey.^9,10^ We used the CHR data for New York state in 2018, which covers 2012 to 2016. The overall heath factors rankings *z* score and each category *z* score for health behaviors, clinical care, social and economic factors, and physical environment were examined in the present study.

### Statistical Analysis

We calculated the correlations between county-level health factors *z* scores and CLRD hospitalization and mortality rates using Pearson *r*. Univariable (UVA) and multivariable linear regression analyses (MVA) were used to determine the associations between county-level health factors *z* score and CLRD hospitalization and mortality rates. Any variables reported in UVA as significantly associated with CLRD outcomes or found to be strongly correlated in the preliminary analyses (Pearson *r* > 0.3 or *r* < −0.3) were then included in the multivariable linear regression model. All data management and statistical analyses were performed in Excel spreadsheets (Microsoft) and SAS statistical software version X.X (SAS Institute)*. P* < .05 using 2-sided *t* tests were considered statistically significant. Data analysis was performed from November 2020 to March 2021.

## Results

### CLRD Hospitalization and Mortality Rates by County in New York State

**Figure 1.**
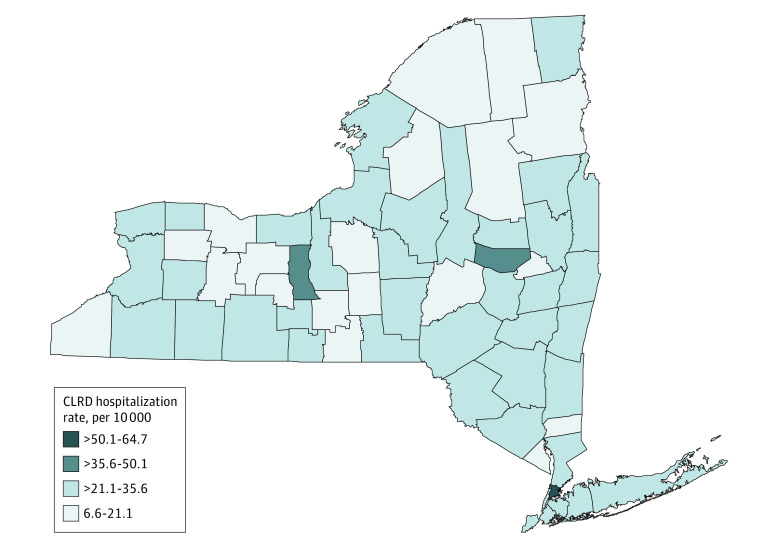
Map of Chronic Lower Respiratory Disease (CLRD) Age-Adjusted Hospitalization Rate per 10 000 Population for 62 Counties of New York State, 2016

**Figure 2.**
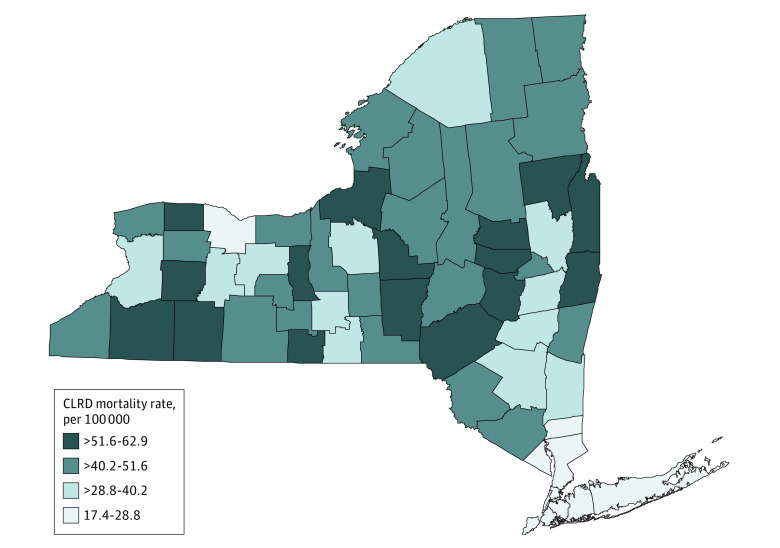
Map of Chronic Lower Respiratory Disease (CLRD) Age-Adjusted Mortality Rate per 100 000 for 62 Counties of New York State, 2014 to 2016

We first examined CLRD statistics in the New York state by analyzing the age-adjusted hospitalization rates in 2016 and mortality rates from 2014 to 2016. During this period, a total of 60 335 discharges were documented as CLRD hospitalizations and 20 612 people died from CLRD in New York state. The overall age-adjusted hospitalization rate was 27.6 per 10 000 population and the overall CLRD mortality rate was 28.9 per 100 000. The analysis based on 62 counties in New York state indicated that Bronx had the highest age-adjusted hospitalization rate (64.7 per 10 000 population) whereas Hamilton had the lowest hospitalization rate (6.6 per 10 000 population). Age-adjusted mortality rates ranged from 17.4 per 100 000 population in Kings to 62.9 per 100 000 population in Allegany. The results are summarized in Table 1 and shown in Figure 1 and Figure 2.

**Table 1.** Summary of County Health Rankings *z* Score and Age-Adjusted Hospitalization and Mortality Rate of 62 Counties in New York State

County	2018 County Health Rankings *z* Score					Age-adjusted rate	
	Overall	Health behaviors	Clinical care	Social and economic factors	Physical environment	Hospitalization, 2016, per 10 000	Mortality rate, 2014-2016, per 100 000
Albany	−0.55	−0.13	−0.14	−0.29	0.006	23.5	34.4
Allegany	0.34	0.11	0.09	0.14	0.003	22.0	62.9
Bronx	1.43	0.08	0.25	1.04	0.049	64.7	25.1
Broome	0.06	0.05	−0.07	0.12	−0.046	23.6	40.3
Cattaraugus	0.55	0.28	0.13	0.12	0.024	29.1	52.5
Cayuga	0.25	0.20	0.03	0.00	0.021	28.9	40.3
Chautauqua	0.48	0.34	−0.06	0.22	−0.029	20.8	44.1
Chemung	0.39	0.23	−0.03	0.23	−0.045	33.6	54.8
Chenango	0.07	0.09	0.00	0.04	−0.053	27.1	60.3
Clinton	0.21	0.19	−0.02	0.05	−0.007	24.3	47.5
Columbia	−0.33	−0.11	0.01	−0.18	−0.052	28.9	40.4
Cortland	0.04	0.05	−0.08	0.05	0.007	18.4	48.3
Delaware	0.35	0.08	0.12	0.21	−0.064	21.8	54.5
Dutchess	−0.53	−0.22	−0.06	−0.29	0.041	26.2	33.3
Erie	0.03	0.06	−0.11	0.00	0.068	23.1	39.4
Essex	−0.15	−0.07	−0.01	−0.03	−0.033	14.0	42.8
Franklin	0.52	0.25	0.02	0.25	−0.005	16.1	50.0
Fulton	0.41	0.12	0.10	0.24	−0.057	24.9	57.4
Genesee	−0.09	0.04	0.05	−0.23	0.042	17.0	42.0
Greene	0.16	−0.07	0.14	0.08	0.006	27.3	36.6
Hamilton	−0.15	−0.13	−0.07	0.15	−0.108	6.6	43.7
Herkimer	0.39	0.20	0.10	0.10	−0.008	24.6	49.6
Jefferson	0.33	0.33	−0.08	0.10	−0.009	24.1	44.2
Kings	0.38	−0.14	0.12	0.38	0.027	30.7	17.4
Lewis	0.25	0.13	−0.03	0.17	−0.021	17.9	41.8
Livingston	−0.13	−0.07	0.04	−0.14	0.046	19.4	37.9
Madison	−0.13	0.02	−0.06	−0.09	0.005	22.8	55.3
Monroe	−0.13	−0.03	−0.06	−0.03	−0.006	20.6	27.1
Montgomery	0.52	0.15	0.01	0.36	0.010	36.1	52.1
Nassau	−1.17	−0.49	−0.16	−0.54	0.019	21.7	20.6
New York	−0.34	−0.31	−0.16	0.12	0.008	24.9	17.9
Niagara	0.37	0.17	0.06	0.11	0.025	34.6	49.6
Oneida	0.19	0.09	−0.02	0.11	0.003	28.2	44.8
Onondaga	−0.23	−0.01	−0.18	−0.05	0.009	20.7	35.9
Ontario	−0.38	−0.06	0.01	−0.32	−0.015	19.7	39.5
Orange	−0.21	−0.09	0.00	−0.17	0.045	31.4	40.5
Orleans	0.47	0.16	0.20	0.10	0.005	21.6	56.9
Oswego	0.61	0.30	−0.03	0.31	0.030	24.6	51.7
Otsego	−0.21	0.00	−0.09	−0.11	−0.013	20.4	42.6
Putnam	−0.82	−0.22	−0.05	−0.56	0.005	17.9	26.4
Queens	−0.02	−0.30	0.23	0.06	−0.007	23.0	18.1
Rensselaer	−0.29	0.01	−0.04	−0.22	−0.034	26.2	55.1
Richmond	−0.13	−0.16	−0.08	0.09	0.025	34.4	28.2
Rockland	−0.6	−0.4	−0.09	−0.19	0.077	18.2	25.8
St Lawrence	0.35	0.13	0.00	0.22	0.014	37.4	52.3
Saratoga	−0.84	−0.14	−0.15	−0.56	0.018	17.4	39.0
Schenectady	−0.18	0.00	−0.12	−0.07	0.020	24.2	39.2
Schoharie	0.02	−0.01	0.03	0.02	−0.007	18.7	40.9
Schuyler	0.31	−0.07	0.17	0.20	0.012	24.5	53.2
Seneca	0.15	0.02	0.09	0.02	0.023	26.0	42.1
Steuben	0.16	0.15	−0.02	0.08	−0.050	21.4	46.0
Suffolk	−0.58	−0.26	−0.01	−0.32	0.002	23.6	28.2
Sullivan	0.33	0.05	0.13	0.18	−0.029	26.4	45.6
Tioga	−0.1	−0.03	0.02	−0.05	−0.040	9.1	36.7
Tompkins	−0.76	−0.21	−0.23	−0.32	0.002	11.0	33.3
Ulster	−0.22	−0.11	0.02	−0.12	−0.018	24.6	33.7
Warren	−0.41	−0.07	−0.23	−0.07	−0.049	25.9	56.8
Washington	0.04	0.09	−0.02	−0.06	0.018	30.5	57.4
Wayne	0.17	0.16	0.17	−0.15	−0.013	28.4	44.4
Westchester	−0.82	−0.47	−0.10	−0.32	0.073	22.8	20.4
Wyoming	0.15	0.10	0.14	−0.11	0.023	25.4	59.8
Yates	0.01	−0.07	0.14	−0.06	0.003	14.2	51.5

### Association Between County Heath Factors *z* Score and CLRD Hospitalization and Mortality Rates

**Figure 3.**
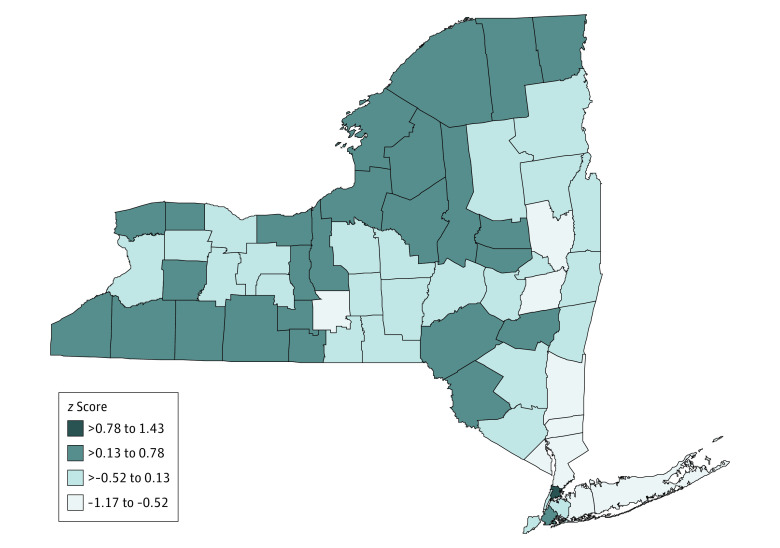
Map of 2018 County Health Rankings (CHR) Overall Health Factors *z* Score for 62 Counties of New York State

Table 1 and Figure 3 show the CHR health factors *z* score for 62 counties in 5 different categories: overall health factors, health behaviors, clinical care, social and economic factors, and physical environment. Among 62 counties, Nassau had the lowest *z* score (the healthiest) for both overall health factors (*z* = −1.17) and health behaviors (*z* = −0.49) and Bronx had the highest *z* score (the least healthy) for overall health factors (*z* = 1.43), clinical care (*z* = 0.25), and social and economic factors (*z* = 1.04).

In Pearson correlation and UVA, counties with higher *z* score of overall health factors (*r* = 0.51; *P* < .001), clinical care (*r* = 0.33; *P* = .008), social and economic factors (*r* = 0.54; *P* < .001), and physical environment (*r* = 0.29; *P* = .02) were correlated with higher CLRD hospitalization rate; given the result of UVA and Pearson correlation, the variables of clinical care, social and economic factors, and physical environment were added to the MVA model, and only social and economic factors (β = 17.61 [95% CI, 10.36 to 24.87]; *P* < .001) and physical environment (β = 82.78 [95% CI, 36.94 to 128.63]; *P* < .001) remained independently associated in MVA (*R^2^* = 0.431). An increase of 1 point of *z* score in social and economic factors was associated with an increase of 17.6 hospitalizations per 10 000 population, and a 1-point increase physical environment was associated with an increase of 82.8 hospitalizations per 10 000 population. Regarding the association between health factors and CLRD mortality rate, counties with higher *z* score of overall health factors (*r* = 0.45; *P* < .001) and health behaviors (*r* = 0.71; *P* < .001) were correlated with higher mortality rate, whereas an opposite association was found in physical environment (*r* = −0.35; *P* = .005). In the MVA model with variables of health behaviors and physical environment, a 1-point increase in *z* score of health behaviors was associated with an increase of 41.4 deaths per 100 000 population (β = 41.42 [95% CI, 29.88 to 52.97]; *P* < .001), and a 1-point increase in physical environment was associated with a decrease of 63.4 deaths per 100 000 population (β = −63.40 [95% CI, −123.86 to −2.94]; *P* = .04; *R^2^* = 0.532). Table 2 summarized the analysis results. We performed an MVA to examine the association between each subfactor of physical environment and CLRD mortality and found that counties with a higher percentage of severe housing problems were significantly associated with lower CLRD mortality (β = −5.92; *P* = .002).

**Table 2.** Linear Regression Analysis of Variables Associated With CLRD Outcomes

Variable	CLRD hospitalization					CLRD mortality				
	Univariable analysis			Multivariable analysis^a^		Univariable analysis			Multivariable analysis^b^	
	*r*	β (95% CI)	*P* value	β (95% CI)	*P* value	*r*	β (95% CI)	*P* value	β (95% CI)	*P* value
										
Health behaviors	0.22	9.56 (−1.46 to 20.58)	.09	NA	NA	0.71	44.35 (32.84 to 55.86)	<.001	41.42 (29.88 to 52.97)	<.001
Clinical care	0.33	24.68 (6.68 to 42.68)	.008	5.69 (−10.96 to 22.35)	.50	0.21	22.45 (−4.38 to 49.28)	.10	NA	NA
Social and economic factors	0.54	17.35 (10.42 to 24.28)	<.001	17.61 (10.36 to 24.87)	<.001	0.22	10.15 (−1.42 to 21.72)	.08	NA	NA
Physical environment	0.29	67.00 (10.43 to 123.57)	.02	82.78 (36.94 to 128.63)	<.001	−0.35	−115.80 (−195.41 to −36.18)	.005	−63.40 (−123.86 to −2.94)	.04
Overall health factors	0.51	9.29 (5.22 to 13.35)	<.001	NA	NA	0.45	11.71 (5.63 to 17.79)	<.001	NA	NA

Abbreviations: CLRD, chronic lower respiratory disease; NA, not applicable.

^a^ *R*^2^ = 0.431.

^b^ *R*^2^ = 0.532.

## Discussion

This cross-sectional study is the first study, to our knowledge, to assess the associations between county-level health factors and CLRD hospitalization and mortality rates in New York state. This study found geographic differences in CLRD outcomes among counties and their associations with county-level health disparities. The mapping of CLRD hospitalization and mortality rates shows the different distribution in New York state. The highest hospitalization rate was found in New York City (Bronx, Kings, New York, Queens, and Richmond counties), whereas the lowest was in North Country (Clinton, Essex, Franklin, Hamilton, Warren, and Washington counties) and Southern Tier (Broome, Chenango, Delaware, Tioga, and Tompkins counties). For mortality, the highest rate was seen in Western New York (Allegany, Cattaraugus, Chautauqua, Erie, Genesee, Niagara, Orleans, and Wyoming counties) and North Country, but the lowest was in New York City.

CLRD hospitalization and mortality rates are associated with cigarette smoking and socioeconomic status (eg, income, education, occupation, respiratory tract infections, air pollution, and housing conditions).^11,12,13,14,15,16,17,18,19,20,21,22,23^ This study found a positive association between overall CHR health factors and CLRD outcomes, indicating higher hospitalization and mortality rates in counties with worse health status. Previous studies have found that SES disparity was associated with the risk of hospitalizations for asthma or COPD. A 2014 study by Trachtenberg et al^24^ reported that patients with CLRD in the lowest income quintile were approximately 3-fold more likely to be hospitalized than those in the highest income quintile. Similarly, studies by Calderón-Larrañaga et al,^25^ McAllister et al,^26^ and Gupta et al^27^ all found a significant association between Index of Multiple Deprivation score, an SES measure using multiple domains (eg, income, housing, access, education), and hospitalizations for asthma and COPD, suggesting lower SES was associated with higher risk of hospitalizations. Interestingly, a 2017 study by Keet et al^28^ also suggested residence in urban or poor areas was an important risk factor for asthma-related emergency department visits and hospitalizations.^28^ These findings are generally consistent with our study result as overall CHR health factors, social and economic factors, and physical environment had significant associations with CLRD hospitalizations in New York state.

Cigarette smoking is a modifiable health-related behavior and is associated with accelerated decline of lung function and higher mortality rates for asthma and COPD.^29^ Previous research identified cigarette smoking as a leading risk factor of increased numbers of life threatening asthma attacks and greater asthma mortality.^30,31,32,33^ A study by Marquette et al^30^ reported that the odds of mortality were 3.6-fold in smokers with asthma compared with nonsmokers with asthma. Smoking cessation is an essential intervention in the treatment of COPD to slow the progressive decline in lung function as well as improve survival in COPD. In the Lung Health Study,^34,35^ 5587 smokers with mild to moderate COPD were randomized to smoking cessation or usual care, and after 14.5 years of follow-up, those who quit smoking had better lung function and a lower mortality rate. Likewise, the 2014 Surgeon General’s Report indicated nearly 8 out of 10 COPD-related deaths were a result of smoking.^36^ All these findings can be the reason that overall CHR health factors and health behaviors were strongly associated with CLRD mortality in New York state.

A surprising and unexpected finding was the paradoxical association between the physical environment indicator and CLRD mortality. The negative association was not consistent with our expectation that better physical environments would improve health outcomes. Therefore, we performed an MVA to examine the association between each subfactor of physical environment and CLRD mortality. The analysis showed counties with higher percentage of severe housing problems were significantly associated with lower CLRD mortality. Lack of a kitchen is one of the main components of severe housing problems, and that could be an explanation for this finding, as several studies suggested exposure to indoor combustion and cooking fuels may increase the risk of asthma and COPD and associated outcomes.^37,38,39^

The CHR is a program to evaluate multiple factors that can impact health disparities and determine the health status of counties in the state. The reliability and accuracy of CHR are important to consider for describing patterns across a state. A report by Lahiri et al^40^ found that North Country in upstate New York and Bronx county in downstate New York both have the worst quality of health and income-related health inequality, suggesting areas with lower quality of health have larger health disparities between the rich and the poor in New York state. These geographic findings are similar to CHR data for New York state. A study by Arndt et al^41^ also found an interesting negative correlation between reliability of CHR measures and number of counties in a state, suggesting that New York state, with 62 counties, may have higher reliability in the CHR measures. Therefore, CHR could be considered a reliable measure to characterize the county health status in New York state.

### Limitations

There are some limitations to our study. The standardized manner of data collection using population-based surveillance to examine CLRD outcomes and county-level health indicators in New York state is a strength. However, the hospitalization data are based on diagnosis *ICD-10-CM* codes^8^ from the hospital data system, and mortality data are based on death certificates from Vital Statistics of New York State Department of Health. There are intrinsic limitations and concerns related to the potential for inconsistent reports leading to misclassification and incorrect coding of diagnoses by physicians in the data set. Using indicators from CHR to represent the health disparities among counties in New York state is another limitation and may not be specific. The data of CHR indicators included in the annual rankings may be from different time frames, such as Behavioral Risk Factor Surveillance System data from 2016 and American Community Survey data from 2012 to 2016 in the 2018 CHR report. Another important limitation in this study is the ecological fallacy, since associations on population levels may not reflect associations on individual levels. Therefore, these results must be interpreted with caution, and further studies are suggested to support our findings.

## Conclusions

This cross-sectional study found a remarkable variation on CLRD hospitalization and mortality rates in New York state. CLRD outcomes were significantly associated with county-level health disparities. These findings suggested that public health interventions and resources aimed at improving CLRD outcomes should be tailored and prioritized in health disadvantaged areas.
